# Pharmacological Properties of the Type 1 Tyramine Receptor in the Diamondback Moth, *Plutella xylostella*

**DOI:** 10.3390/ijms20122953

**Published:** 2019-06-17

**Authors:** Haihao Ma, Qingting Huang, Xiaoyi Lai, Jia Liu, Hang Zhu, Yong Zhou, Xile Deng, Xiaomao Zhou

**Affiliations:** Institute of Agricultural Biotechnology, Hunan Academy of Agricultural Sciences, Changsha 410125, China; mhh8486@163.com (H.M.); hqting93@163.com (Q.H.); laixiaoyi@hnu.edu.cn (X.L.); jialiuv@126.com (J.L.); zhyhhnl@163.com (H.Z.); qimiaobuchugan@126.com (Y.Z.); chemdxl@163.com (X.D.)

**Keywords:** tyramine receptor, *P. xylostella*, agonist, antagonist, gene expression

## Abstract

Tyramine receptors (TARs) can be activated by tyramine (TA) or octopamine (OA) and have been shown to be related to physiological regulation (e.g., gustatory responsiveness, social organization, and learning behavior) in a range of insect species. A tyramine receptor gene in *Plutella xylostella*, *Pxtar1*, was cloned and stably expressed in the HEK-293 cell line. Pharmacological properties and expression profile of *Pxtar1* were also analyzed. Tyramine could activate the PxTAR1 receptor, increasing the intracellular Ca^2+^ concentration ((Ca^2+^)i) at an EC50 of 13.1 nM and reducing forskolin (10 μM)-stimulated intracellular cAMP concentration ((cAMP)i) at an IC_50_ of 446 nM. DPMF (a metabolite of amitraz) and L(-)-carvone (an essential oil) were found to act as PxTAR1 receptor agonists. Conversely, yohimbine and mianserin had significant antagonistic effects on PxTAR1. In both larvae and adults, *Pxtar1* had the highest expression in the head capsule and expression of *Pxtar1* was higher in male than in female reproductive organs. This study reveals the temporal and spatial differences and pharmacological properties of *Pxtar1* in *P. xylostella* and provides a strategy for screening insecticidal compounds that target PxTAR1.

## 1. Introduction

Norepinephrine, epinephrine, and catecholamine regulate important physiological functions in vertebrates. In insects, these biochemicals are replaced by TA and OA [1]. TA is a precursor for the synthesis of OA and can cooperate with OA to regulate multiple insect behaviors (e.g., fight or flight [2], olfactory response [3], and fertilization [4]). TA also has physiological roles independent of OA in the nervous system [5]. For example, TA inhibits serotonin (5-HT)-stimulated pharyngeal pumping in *Caenorhabditis elegans* [6], modulates locomotion pattern generators in *Drosophila melanogaster* [7], and *Manduca sexta* [8] larvae, suppresses pheromone production in *Bombyx mori* [9], acts as a diuretic factor in *D. melanogaster* [10], regulates repulsive behavior of *Locusta migratoria* [11], and regulates honeybee gustatory responsiveness, social organization, and learning behavior [12]. As an important signaling molecule, abnormal TA levels can seriously affect the physiological activities of insects. Indeed, in a study Vanden found that repeated TA injections could reduce the viability of last instar *L. migratoria* and *Schistocerca gregaria* larvae [13].

In insects, TA functions as a regulatory molecule by specifically binding to either tyramine receptors (TARs) or octopamine receptors (OARs) [14]. Both TARs and OARs are G protein coupled receptors. Two types of TARs have been identified in lepidopteran insects and a majority of research has focused on the type 1 tyramine receptor (TAR1). In previous studies, TAR1 expression was evaluated in many pests, such as *Agrotis ipsilon* [15], *Chilo suppressalis* [16], *Periplaneta americana* [1,17], etc., while the tissue specific expression of TAR1 in *D. melanogaster* is the most well studied [18,19,20,21]. In the larval stages, the *tar1* gene of *D. melanogaster* (*Dmtar1*) was highly expressed in the head, peripheral nervous system, primary branches of the tracheal system, dorsal aorta, salivary glands, and the spiracular openings, and weakly expressed in the Malpighian tubules [20,21]. In adults, *Dmtar1* was highly expressed in abdominal muscles, leg sensilla, ocelli, proboscis, labellum, and central nervous systems (CNS) [19]. Therefore, tissue distribution of TAR1 in insects coincides with the physiological functions of TA, making TAR1 an important candidate target for pest control products.

Most TAR1 proteins can be activated by both TA and OA; however, TA has higher activity than OA [14]. Activated TAR1 can couple to Gi (a heterotrimeric G protein subunit that inhibits the production of cAMP from ATP), reducing forskolin-stimulated intracellular cAMP levels, and/or couple to Gq (a G protein subunit that activates membrane-bound phospholipase C, which then cleaves PIP2 into IP3 and diacylglycerol), increasing intracellular Ca^2+^ concentrations [14]. Both (Ca2+)i and (cAMP)i assays represent effective tools to study the pharmacological properties of TAR1. TAR1 represents a good potential candidate for use in production of various pest control products. Firstly, recent studies have found that formamidine insecticides (such as chlordimeform and amitraz) can specifically target this receptor [22,23]. Secondly, TAR1 mutation has been associated with pesticide resistance in ticks [24,25]. Finally, some efficacious insecticidal essential oils (e.g., L(-)-carvone, thymol, p-cymene) have been found to specifically interact with this receptor [26,27]. Therefore, the study of the pharmacological properties of TAR1 can help us understand the molecular interactions between receptor proteins and pesticides, and can provide an effective method for high-throughput drug screening and rational drug design based on TAR1.

*Plutella xylostella* (diamondback moth) is an important pest of vegetable crops. In China, approximately 0.77 billion dollars are expended to control these pests each year, and much research has focused on their biology and potential management strategies [28]. As a potential pesticide target, the biological function of TARs in *P. xylostella* is still unclear. In this study, we have cloned the *Pxtar1* gene, generated a HEK-293T cell line capable of stably expressing TAR1 (TAR1/293T), and analyzed the pharmacological properties of this receptor using (Ca^2+^)i and (cAMP)i assays. Furthermore, we have investigated the temporal and spatial expression of the *Pxtar1* gene both in adults and larvae of *P. xylostella*. This research will elucidate the physiological function of PxTAR1 protein and can potentially be applied to develop or screen pest control products that can be used in *P. xylostella* management.

## 2. Results

### 2.1. cDNA Cloning and Sequences Analysis of PxTAR1

The *P. xylostella tar1* gene (*Pxtar1*) was made up of 1404 base pairs, and the expressed PxTAR1 protein has 467 amino acids (AAs, Figure S1), with molecular weight of 52266 Daltons. BLAST analysis of PxTAR1 receptor showed that PxTAR1 sequences had an 82% similarity with *B. mori* TAR1 and an 81% similarity with *C. suppressalis* TAR1. Other Lepidoptera TAR1 protein sequences had 80–85% similarities to the deduced PxTAR1 protein (Table S1).

**Figure 1:**
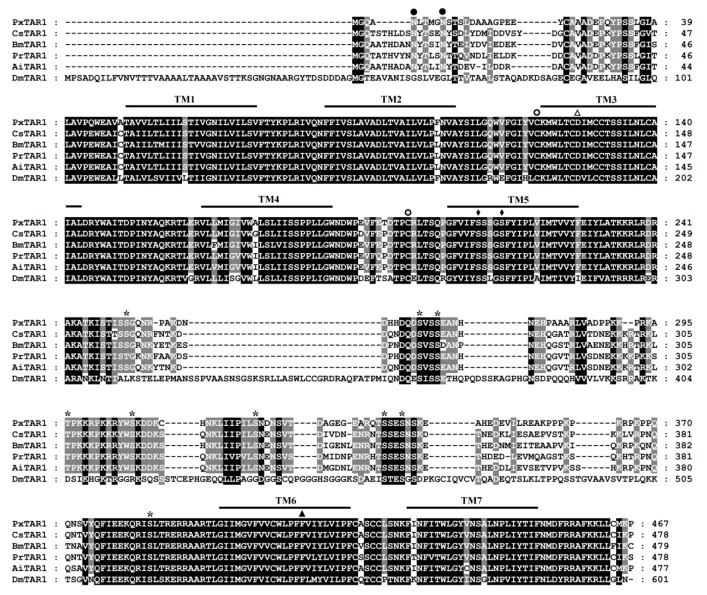
Multiple sequence alignment of PxTAR1 and orthologous receptors. Shaded sequences highlight the identity level of amino acids between the receptors. Amino acid position is indicated on the right and the predicted seven transmembrane regions are indicated by TM1-7. Potential N-glycosylation sites and potential phosphorylation sites for protein kinase C are labeled by filled circles and asterisks, respectively. Conserved cysteine residues in the first and second extracellular loops are labeled with empty circles. The aspartic acid residue (D127) and the serine residues (S211and S215) that were predicted to be involved in agonist binding are labeled with triangle and filled rhombus. The second phenylalanine after the FxxxWxP motif in TM6, which is a unique feature of aminergic receptor, is indicated by a filled triangle. CsTAR1 (*C. suppressalis*; GenBank accession number: AFG26689), BmTAR1 (*B. mori*; BAD11157), PrTAR1 (*Pieris rapae*; AFX62896), AiTAR1 (*A. ipsilon*; ACN12797), and DmTAR1 (*D. melanogaster*; BAB71788).

Protein transmembrane analysis showed that the PxTAR1 protein has seven transmembrane helical regions (each region was 23 AA long), and the N-terminal of the protein was located at the outer side of the cell and the C-terminal was located at the other side. There were two cysteines located at the extracellular ring between TM2 and TM3 and TM4 and TM5, presumably functioning in the formation of disulfide bonds to enhance protein stability. There were also two N-glycosylation sites in the TAR1 N-terminal, which may play a role in assisting protein folding and regulating protein activity. Residues D127 in TM3 and S211 and S215 in TM5 were detected, likely functioning in substrate binding, and this has also been seen in silkworms [29]. The intracellular loop between TM5 and TM6 was 168 AAs long, and contained several PKC recognized sites, which are necessary for the interaction of TAR1 with G proteins [15]. This intracellular loop also contained a conserved FxxxWLPFF motif in TM6, which is considered essential for bioamine binding [29] (Figure 1).

**Figure 2:**
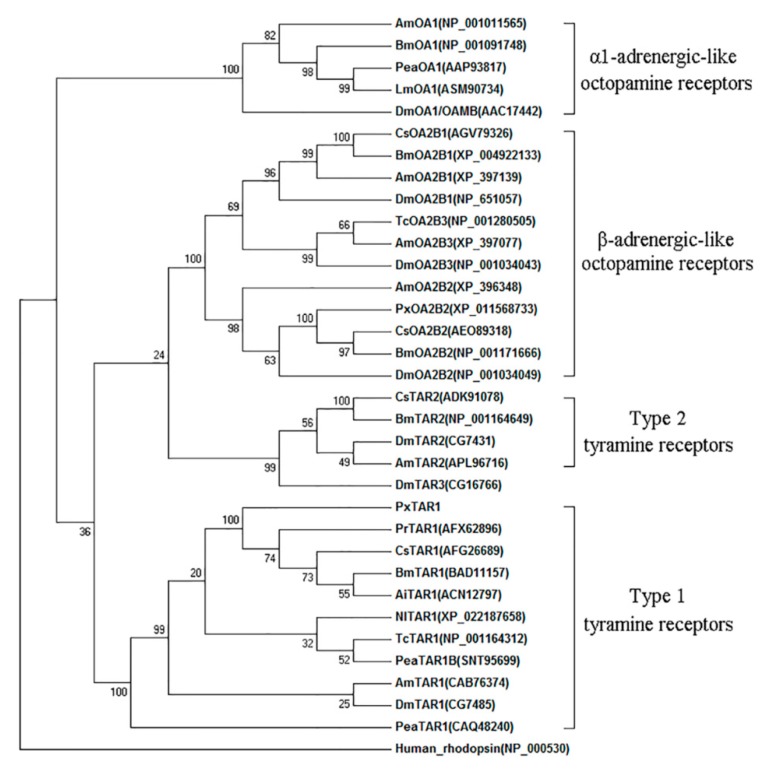
Phylogenetic tree analysis of representative members of the different TARs and OARs. Alignment was performed using amino acid sequences from TM1 to TM7 and the numbers at the nodes of the branches represent the level of bootstrap support for each branch. GenBank accession numbers of each receptor sequences are listed in the tree. Abbreviations of species in alphabetical order are: Ai, *Amphibalanus improvises*; Am, *Apis melifera*; Bm, *Bombys mori*; Cs, *C. suppressalis*; Dm, *D. melanogaster*; Dp, *Danaus plexippus*; Lm, *L. migratoria*; Nl, *Nilaparvata lugens*; Pea, *P. americana*; Pr, *P. rapae*; Px, *P, xylostella*; and Tc, *Tribolium castaneum.*

Phylogenic tree analysis showed that insect TARs have two divergent clades, as the deductive PxTAR1 protein is on the same branch as the insect TAR1, and not clustered with the insect TAR2 or OARs (Figure 2).

### 2.2. Expression Pattern of PxTAR1

**Figure 3:**
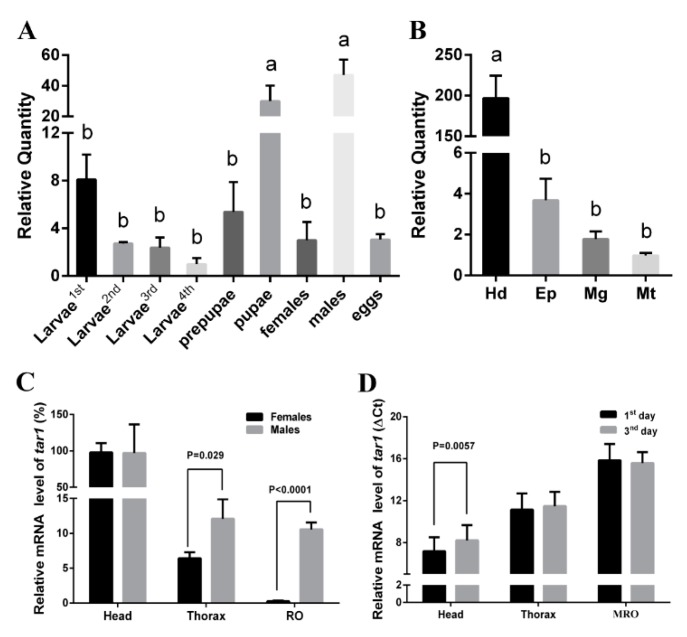
Relative expression (mean ± SE) of *tar*1 in *P. xylostella*. (**A**) Expression of *Pxtar1* genes in different development stages (first to fourth larvae, pre-pupae, pupae, adults, and eggs). *Pxtar1* in fourth larvae was used as a comparator. (**B**) Expression of *Pxtar1* in tissues (the head (Hd), epidermis (Ep), midgut (Mg), and Malpighian tubules (Mt)) of the fourth instar larvae. *Pxtar1* in Mt was used as a comparator. (**C**) Expression patterns of *Pxtar1* in adult tissues (head, thorax, female reproductive organs (FROs) and male reproductive organs (MROs)), *Pxtar1* in FRO was used as a comparator. (**D**) Expression of the *Pxtar1* in female adults in the head and MRO at first and third days. Data were mean values of three independent experiments (*n* = 3) and normalized to endogenous ribosomal protein L32, the internal control

The *Pxtar1* gene was expressed in all developmental stages of *P. xylostella* including larva, pre-pupa, pupa, adult, and egg. The levels of *tar1* mRNA were low in both egg and larval stages, higher in pupal stages, and highest in the adult males (*n* = 3, Figure 3A). Interestingly, there was no significant difference in *tar1* gene expression between developing males and females during the pupal stage, but *tar1* expression was significantly increased in adult males and significantly decreased in the adult females (*p* = 0.002, *n* = 3; Figure S2).

At the fourth instar larval stage, *Pxtar1* was expressed in all tested tissues: it was highly expressed in the head, followed by the epidermis, and then the Malpighian tubule (Figure 3B). Furthermore, *Pxtar1* was highly expressed in the head, followed by the thorax. There were no significant differences in *Pxtar1* mRNA expression in females compared to males, but the *tar1* mRNA levels in female reproductive organs (FROs) were lower than in males (MROs) (*p* < 0.0001, *n* = 3; Figure 3C). Upon comparison of *tar1* expression in the heads, thoraxes, and reproductive organs of adult males on the first and third day, it was found that there were no significant differences in *tar1* levels in the thorax and MRO, but the expression of *tar1* in the head on the third day was significantly lower than that on the first day (*p* = 0.0057, *n* = 3; Figure 3D).

### 2.3. Functional Characterization of PxTAR1 with the Ca^2+^ Assay

**Figure 4:**
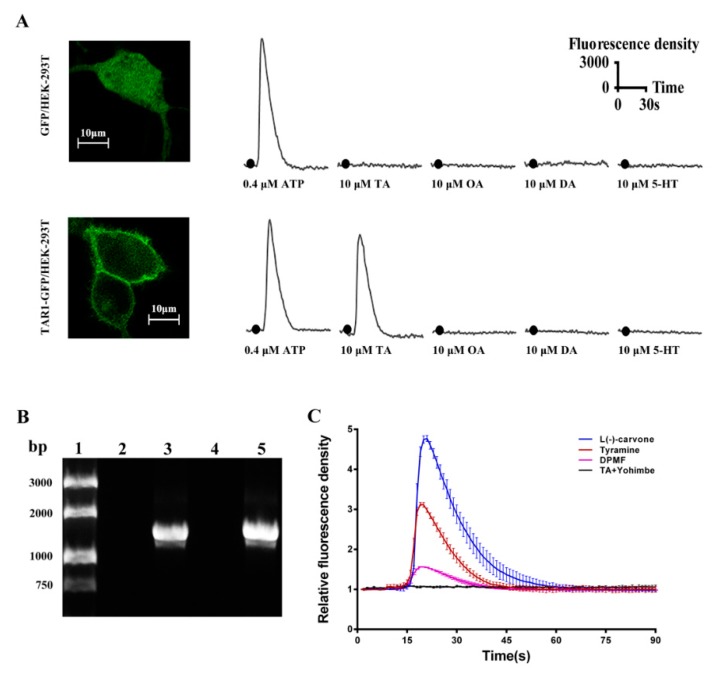
Effects of different reagents (biogenic amines and putative agonists) on (Ca^2+^)i in PxTAR1 over-expressing cells. (**A**) Effects of biogenic amines on intracellular calcium concentration in HEK-293T cells. (**B**) RT-PCR amplification of *tar1* gene using total RNA extracted from GFP/293T and TAR1/293T cells. Lane 1, DNA marker; lane 2, HEK-293 cells stable expression of GFP protein (negative control); lane 3, HEK-293 cells’ stable expression of PxTAR1 protein; lane 4, total RNA from TAR1/293T cells as template; lane 5, positive control (pEGFP-N1-PxTAR1 plasmid). Expected amplification fragment was 1404 base pairs. (**C**) Effects of different agonists on (Ca^2+^)i in TAR1/293T cells. Data are presented as the mean of at least three independent experiments. Relative fluorescence density was defined as: real-time fluorescence density/background fluorescence density.

PxTAR1-GFP fusion proteins were mainly localized in membrane, and GFP protein was diffusely localized across the whole cell (Figure 4A). Common biogenic amines including TA, OA, dopamine (DA), and serotonin (5-HT) (the final concentration was 10 μM) were used to examine the functional properties of the PxTAR1 in HEK-293 cells. TA could elicit Ca^2+^ responses at concentrations of 10 μM, OA could elicit Ca^2+^ responses at concentrations of 100 μM, (Figure S3) and no other compounds elicited Ca^2+^ expression at 10 μM concentrations (Figure 4A). This suggests that the PxTAR1 was selectively activated by TA and activation was coupled with intracellular Ca^2+^ mobilization.

A HEK-293 cell line stably expressing PxTAR1 was generated and identified by reverse transcription PCR with specific primers (Figure 4B). Several OARs agonists (such as clonidine, naphazoline, phenylethylamine, N2-(2,4-Dimethylphenyl)-N1-methyformamidine (DPMF)), antagonists (including phentolamine, mianserin), and plant essential oils (L(-)-carvone, thymol, anethole, and eugenol) were used to treat the HEK-293 cells stably expressing PxTAR1 at 10 μM concentrations. Compared to control cells stably expressing GFP protein, only L(-)-carvone and DPMF could elicit Ca^2+^ responses at concentrations of 10 μM. L(-)-carvone strongly stimulated PxTAR1 and elicited a Ca^2+^ response with EC_50_ of 1.15 nM, and DPMF (EC_50_ = 84.5 nM) had a weaker activity than TA (EC_50_ = 13.1 nM, Figure 4C and Figure S3). Yohimbine (reported as an antagonist of TARs) was tested, and it was found that this compound could completely block the TA-stimulated increase of (Ca^2+^)i at a concentration of 10 μM (Figure 4C). Other tested regents did not show distinct agonistic or antagonistic efficiency (data not show).

### 2.4. Pharmacological Characterization of PxTAR1 Based on cAMP Assay

**Figure 5:**
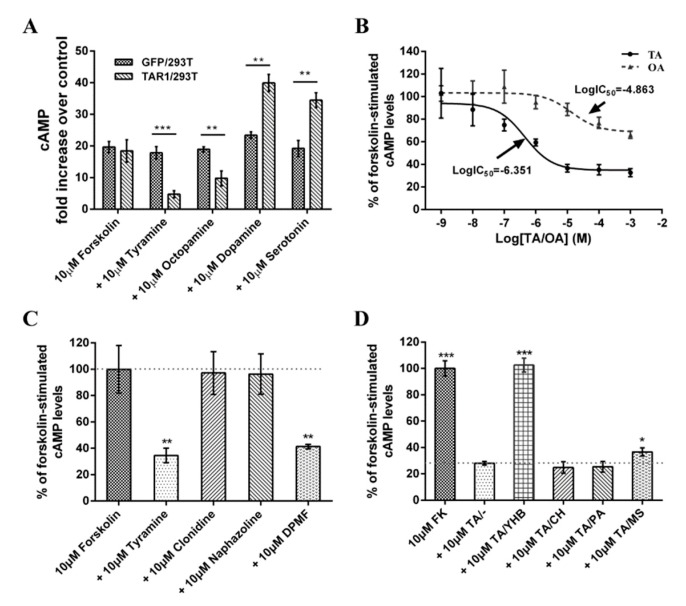
Regulation of (cAMP)i in HEK-293T cells stably expressing the PxTAR1 receptor (TAR1/293T) compared to GFP/293T cells (negative control). (**A**) Regulation of forskolin and biogenic amines on (cAMP)i. (**B**) Dose–response analysis of TAR1/293T cells to TA and OA and attenuation of forskolin-stimulated intracellular cAMP levels. (**C**) Regulation of putative agonists (clonidine, naphazoline, and DPMF) on forskolin-stimulated cAMP levels in TAR1/293T cells. (**D**) Regulation of putative antagonists on TA attenuation of forskolin-stimulated intracellular cAMP levels. Abbreviations: TA/–, tyramine and no antagonist; TA/YHB, tyramine + yohimbine; TA/CH, tyramine + chlorpromazine; TA/PA, tyramine + phentolamine; TA/MS, tyramine + mianserin. Data are presented as means ± SE of three experiments (*n* = 3). Statistically significant differences: *** *p* ≤ 0.001, ** *p* ≤ 0.01, * *p* ≤ 0.05.

Application of 10 μM of forskolin stimulated adenylyl cyclase, which increased (cAMP)i levels in both TAR1/293T cells and in GFP/293T cells (Figure 5A). 10 μM OA and TA reduced forskolin (10 μM)-stimulated intracellular cAMP levels by approximately 47.1 and 74.2% in TAR1/293T cells (not GFP/293T cells) (*n* = 3, Figure 5A). Dopamine and serotonin were also tested, and results showed that dopamine produced 2.17 fold increases in forskolin-stimulated intracellular cAMP levels in PxTAR1-expressing cells and serotonin produced 1.87 fold increases in forskolin-stimulated intracellular cAMP levels in the TAR1/293T cells (Figure 5A), suggesting that dopamine and serotonin could activate the endogenous cAMP pathway, and PxTAR1 could enhance this effect.

To further investigate PxTAR1′s properties, concentration–response curves for TA and OA were established (concentration range from 10^−9^ M to 10^−3^ M). The half maximal reduction in cAMP levels (IC_50_) was 446 nM (95% confidence intervals, 150 nM–1325 nM, *n* = 3) for TA and 13.7 μM (95% confidence intervals, 3.47 μM~53.9 μM, *n* = 3) for OA. TA was more effective at activating TAR1 than OA (~30 fold, *p* = 0.0037) (Figure 5B).

Three agonists (naphazoline, clonidine, and DPMF) were tested, only DPMF reduced forskolin (FK, 10 μM)-stimulated intracellular cAMP levels in TAR1/293T cells at a concentration of 10 μM (Figure 5C). Furthermore, TA-induced cAMP induction could be blocked by treatment with yohimbine (10 μM, *p* < 0.0001, *n* = 3) or mianserin (10 μM, *p* = 0.012, *n* = 3), but not chlorpromazine and phentolamine (Figure 5D).

## 3. Discussion

In insects, TA regulates important physiological activities by activating TARs or OARs [14]. Compared to OARs, TARs have a higher affinity for TA [1,16,30,31]. As an important receptor protein in the nervous system, TAR1 has been cloned and studied in bees [32], fruit flies [21], nematodes [33], locusts [34,35], silkworms [31], and ticks [22,23]. In this study, we cloned the TAR1 receptor gene of *P. xylostella* for the first time. Phylogenetic tree analysis showed that PxTAR1 was strongly clustered with BmTAR1, CsTAR1, PrTAR1, and AiTAR1, and thus, likely belonged to the TA receptor 1 family (Figure 2). Four biogenic amines including OA, TA, dopamine, and serotonin (at 10 μM) were used to treat HEK-293 cells stably expressing PxTAR1. It was found that only OA and TA could significantly reduce the forskolin-stimulated intracellular cAMP level. TA was more potent than OA (TA IC_50_ = ~446 nM, OA IC_50_ = ~13.7 μM), similar results have also been reported in AmTAR1 (TA IC_50_ = ~130 nM, OA IC_50_ = ~3 μM) [32], PeaTAR1 (TA IC_50_ = ~350 nM) [17], and CsTAR1 (TA IC_50_ = ~369 nM) [16]. Cal-590^TM^ fluorescent dye was used to determine the effects of the four biogenic amines on (Ca^2+^)i in TAR1/293T cells. It was found that only TA could elicit significant changes of (Ca^2+^)i at 10 μM (Figure 4A). These characteristics of PxTAR1 conform to the taxonomic characteristics of TAR1 receptors. Therefore, based on our comprehensive analysis, the TAR1 receptor gene of *P. xylostella* was successfully cloned in this study.

Tissue localization analysis provided a key to understanding of the function of TARs in *P. xylostella*. We found that the expression of PxTAR1 increased several times at the stages from pupa to adult and was more abundantly expressed in the head than that in the body. Similar expression patterns of TAR1 were found in *B. mori*, *D. melanogaster,* and *C. suppressalis* [16]. These results suggest that PxTAR1 plays a similar role to orthologous proteins in insects, such as processing sensory information [3], regulating feeding behaviors and/or social behaviors [11,12]. There are not very many studies focused on the expression differences between males and adult females. Here, we found that TAR1 expression level in adult males was 15.6 fold higher than that in adult females. Furthermore, expression in both thoraxes and reproductive organs of adult males were significantly higher than that in adult females, suggesting that PxTAR1 plays a role in modulating the sexual behaviors of males. However, this detailed regulation process needs further study. Interestingly, expression of *Pxtar1* in adult males was downregulated on the third day after eclosion. Typically, adult males begin to mate on the second day after eclosion; however, in this study peak mating occurred on the third day, suggesting that PxTAR1 regulated courtship activity as an inhibitory neuromodulator. Similar results have been reported in *D. melanogaster* [36].

TARs have important biological functions. Therefore, the pharmacological properties of TARs have always been a research hotspot. Four agonists and four antagonists were used to perform pharmacological characterization of PxTAR1 in TAR1/293T cells. Clonidine, naphazoline, amitraz, and DPMF are agonists of beta-adrenergic-like octopamine receptor (PxOA2B2) in *P. xylostella* [37]. Here, we tested the effects of these regents on PxTAR1 and found that clonidine and naphazoline could not activate PxTAR1. Similar results have been reported in the studies of TAR1 from *B. mori* [31,38] and *C. suppressalis* [16]. Amitraz can significantly reduce (cAMP)i production in the HEK-293 cells stably expressing CsTAR1. However, we found that amitraz could not increase (Ca^2+^)i in TAR1/293T cells at 10 μM, but DPMF (a metabolite of amitraz) could active PxTAR1 and induce Ca^2+^ response and reduce the forskolin-stimulated intracellular cAMP level as TA. Therefore, DPMF, not amitraz, is an effective agonist of PxTAR1. *R. microplus* TAR1 (RmTAR1) is considered a likely target of formamidine pesticides (e.g., amitraz and chlordimeform) [24]. Whether PxTAR1 can be used as a target for formamidine pesticides like RmTAR1 needs further study. Phentolamine, chlorpromazine, mianserin, and yohimbine are reported TAR antagonists. In this study, yohimbine had a strongly antagonistic effect on PxTAR1 and mianserin had a weakly antagonistic effect on PxTAR1 (Figure 5). Unlike TAR1 of *C. suppressalis* [16], *B. mori* [38] and *P. americana* [1], chlorpromazine, and phentolamin did not have significant antagonistic effects on PxTAR1 in cAMP assays. This result may be due to the slight differences in the structures of TAR1 protein in different species.

Some plant essential oils have been reported to activate TARs or OARs in previous studies [26,27]. For example, L(-)-carvone-treated DmTAR1-expressing cells induced a significant decrease of (cAMP)i and a remarkable increase of (Ca^2+^)i; thymol can bind to DmTAR1 and is a toxic chemical against wild-type *D. melanogaster* strain (LD_50_ = 0.9 μg/fly); eugenol decreased cAMP level in HEK-293 cells expressing alpha-adrenergic-like OARs of *P. americana*; trans-anethole increased cAMP in HEK-293 cells expressing beta-adrenergic-like OARs of *D. melanogaster*. Exploring the activation of plant essential oils on TARs or OARs will help us to understand the mechanism of these chemicals in pest control. Here, we tested them using TAR1/293T cells by (Ca^2+^)i assay, and found that only L(-)-carvone could induce a significant increase in (Ca^2+^)i. This result is similar to what has been previously seen with *D. melanogaster* [26] and whether L(-)-carvone regulates the physiological or behavioral processes of *P. xylostella* needs further study.

## 4. Materials and Methods

### 4.1. Insect and Chemicals

*P. xylostella* eggs were purchased from Keyun Biocontrol (Jiyuan, Henan, China) and raised in the insectarium of the Hunan Agricultural Biotechnology Institute under the following conditions: 25 ± 1 °C, 65% ± 5% relative humidity, and a photoperiod of 16L:8D. The larvae were fed with cabbage leaves and the adults were fed with a 5% solution of honey in water. The RNA extractions for cloning of *tar1* were from the second day of fourth instar larvae. Gene expression profile analyses were performed with fourth instar larvae and adults.

Chemicals and reagents were purchased from Invitrogen (Carlsbad, CA, USA), AAT Bioquest (Sunnyvale, CA, USA), Sigma–Aldrich (St. Louis, MO, USA), and Vazyme Biotech (Nanjing, Jiangsu, China) if their origins were not mentioned in the text. N2-(2,4-Dimethylphenyl)-N1- methyformamidine (DPMF) [39] was synthesized by Shenyang Research Institute of Chemical Industry Co., Ltd. (Shenyang, Changchun, China). Chemicals were diluted in DMSO (Sigma-Aldrich), and then diluted in Hank’s Balanced Salt Solution (HBSS, Solarbio, Beijing, China). The final concentration of DMSO used in experiments (exposed to cells) was 0.1%.

### 4.2. Cloning of PxTAR1 cDNAs

Total RNA was extracted with Total RNA Extraction Reagent (Vazyme Biotech) following the manufacturer’s instructions. The 1st strand cDNA was synthesized from 1 μg RNA using a HiScript 1st Strand cDNA Synthesis Kit (Vazyme Biotech) and stored at −20 °C until use. Using the transcriptome shotgun Assembly database (taxid: 51655), three partial sequences (GenBank: GBAC01017762, GBAC01033030, GBAC01031396) of candidate *Pxtar1* genes were found via homologous alignment of the orthologous genes sequences of *tar1* from *B. mori* and *C. suppressalis* (GenBank accession number: AB162828 and JQ416145). Missing nucleotide sequences were obtained from the whole-genome shotgun contigs database (taxid: 51655) by nucleotide BLAST analysis.

Specific primers, TAR1-HindIII-F and TAR1-PstI-R, were designed to amplify the complete open reading frame (ORF) of the PxTAR1 (Table 1). PCR was completed in a 50 μL reaction system, containing 4 μL of cDNA, 2 μL of each specific primer, 25 μL of 2× Phata Max buffer, and 1 μL of Phanta superfidelity DNA polymerase (Vazyme Biotech). Reaction conditions were as follows: predenaturation at 95 °C for 3 min, followed by 35 cycles at 95 °C for 15 s, 60 °C for 15 s, and 72 °C for 90 s. The final extension was carried out at 72 °C for 5 min. PCR products were separated and subcloned into a pEFGP-N1 vector with DNA restriction endonucleases HindIII and PstI. The ligation product (pEGFP-N1–PxTAR1) was transformed into *E. coli* DH5α-competent cells. Positive clones were selected using Luria–Bertani broth agar plates with 50 μg/mL kanamycin. Plasmids were then extracted and verified by sequencing. *Pxtar1* ORF sequences were obtained by PCR amplification and submitted to GenBank database (Accession Number: MK166023).

### 4.3. Sequence Analysis and Phylogenic Tree Construction

The alignment of putative *Pxtar1* ORFs with relative genomic sequences was performed using NCBI BLAST (https://blast.ncbi.nlm.nih.gov/Blast.cgi) and the exon and intron architectures of *Pxtar1* were analyzed. Multiple sequence alignments of PxTAR1 with orthologous proteins were performed with the ClustalW2 program (https://www.ebi.ac.uk/Tools/msa/clustalw2/). The transmembrane helices in PxTAR1 were predicted by the TMHMM 2.0 online service (http://www.cbs.dtu.dk/services/TMHMM/). Potential PKC phosphorylation sites and N-glycosylation sites were predicted using the Group-Based Prediction System (GPS3.0, http://gps.biocuckoo.org/). Potential palmitoylation sites of the conserved cysteine residues were predicted using GPS-lipid v1.0 software (http://lipid.biocuckoo.org/). Phylogenetic trees were constructed using MEGA 7 software (https://www.megasoftware.net/) using the neighbor-joining method and bootstrapped with 1000 replications.

### 4.4. Gene Expression Profile Analysis

Total RNAs were isolated from *P. xylostella* samples at various developmental stages (included eggs, 1st to 4th instar, pupae, adult females, and adult males.) or different tissues (samples including the head, midgut, Malpighian tubule, epidermis were dissected from 4th instar and the head, thorax, reproductive organs of abdomen from adults) in 4th instar larvae and adults using the Total RNA Extraction Reagent (Vazyme Biotech) following the manufacturer’s instructions. For analysis of different developmental stages, samples of different tissue were made and first-strand cDNA was synthesized with HiScript Q RT SuperMix with gDNA wiper (Vazyme Biotech), using either random primers or the Oligo dT primer mix, and 0.5 μg total RNA template in a 10 μL reaction system. FastStart Essential DNA Green Master (Roche, Pleasanton, CA, USA) was used for Real-time qPCR analysis, which was performed to characterize the relative expression of *Pxtar1* in the different samples using ribosomal protein L32 (*rpl32*) as the internal reference gene [37]. The primers used for real-time qPCR are listed in Table 1. Reaction conditions were as follows: predenaturation at 95 °C for 10 min, followed by 45 cycles at 95 °C for 10 s, 59 °C for 10 s, and 72 °C for 15 s. Relative expression was quantified using the 2^−ΔΔ*C*T^ method [40].

### 4.5. Cell Transfection and Creation of Stable Cell Lines

The HEK-293 cells were grown in Dulbecco’s Modified Eagle Medium (DMEM, 4.5 g/L glucose), and transfected using a lipofectamine 2000 transfection reagent [37]. Briefly, HEK-293 cells were inoculated in six-well cell culture plates one day prior to transfection at a cell density of 0.8 × 10^6^/well, then, during their exponential growth phase, the cells were transfected with pEGFP-N1 or pEGFP-N1-PxTAR1 plasmids (4 μg DNA/ well). After transfection, cells were digested with trypsin and re-inoculated in Nunc™ Glass Bottom Dishes (35 mm dish) for the confocal imaging (Figure 4A).

The cell line stably expressing TAR1 (TAR1/293T) was used for Ca^2+^ assay and cAMP assay. The coding sequence for *Pxtar1* was generated via PCR using specific primers (Table 1), then was digested using XbaI and SalI, and subcloned into a pEB-GFP(T2A)PURO vector (FITGENE, Guangzhou, China), yielding a pEB-TAR1-3flag-copGFP(T2A)PURO reconstructed lentivirus vector which can express both TAR1 and GFP proteins. Packaging of the TAR1-overexpressing lentivirus and selection of the stable-expression cell lines were performed by the FitGene BioTechnology Company (Guangdong, Guangzhou, China). Briefly, the pEB-TAR1-3flag-copGFP(T2A)PURO vector and two helper vectors, pSPAX2 and pMD2G, were co-transfected into the 293T cell line, yielding the TAR1-overexpressing lentivirus. Then the 293T cell line was infected with the lentivirus and stably transfected cells were selected via the addition of 1 μg/mL of puromycin (Sigma–Aldrich, St. Louis, MO, USA) to the medium. The control cell line, GFP/293T, which stably-expressed the GFP protein, was purchased from FitGene BioTechnology Company.

### 4.6. Ca^2+^ and Cyclic AMP (cAMP) Assays

Intracellular Ca^2+^ was measured using Cal-590™ AM (AAT Bioquest), which is a fluorescein-based Ca^2+^ indicator with BAPTA fused into the xanthene fluorophore of fluorescein. This indicator has a Ca^2+^ binding affinity of Kd = 561 nM. The single-photon excitation and emission peak wavelengths of Cal-590™ are 570 and 590 nm, respectively [41]. The HEK-293 cells stably expressing PxTAR1 were seeded on a 35 mm glass bottom dish (NEST, Wuxi, Jiangsu, China) with 2 mL of DMEM supplemented with 10% FBS and incubated for 24 h at 37 °C and 5% CO_2_, at a density of 8 × 10^5^ cells/dish. Half of the medium was discarded and Cal-590™ AM was prepared in 20 µM in Hank’s and Hepes buffer (HHBS) with 0.04% Pluronic^®^ F-127. 1 mL of the working dye solution was added to each culture dish. The dye-loading plate was incubated in a cell incubator for 60 to 90 min, and then incubated at room temperature for another 30 min. The dye working solution was replaced with HHBS containing 1 mM probenecid to remove excess probes. Cytoplasm calcium density was measured using an LSM 880 laser scanning confocal microscope (ZEISS, Oberkochen, Germany). Cal-590™ was excited at 514 nm and confocal imaging was taken every 1 s for 180 s. Fluorescence density was analyzed using Image-Pro Plus 6.0 (Media Cyberneticsy, Silver Spring, Maryland, USA) and data were analyzed using Prism software (Version 6.0, GraphPad, San Diego, CA, USA).

The cAMP assay was performed using the cAMP Parameter Assay Kit (R&D Systems, Minneapolis, MN, USA) as previous described [37]. Briefly, TAR1/293T or GFP/293T cells were plated onto 24 well tissue culture plates (NEST, Jiangsu, Wuxi, China) with DMEM supplemented with 10% FBS at a density of 4 × 10^5^ cells/well. The culture plates were then incubated for 1 day. Then cells were washed in phosphate buffered saline (PBS, pH = 7.4) and equilibrated for 20 min at 37 °C in 100 μM of the phosphodiesterase inhibitor 3-isobutyl-1-methyl xanthine (IBMX). Two-hundred microliters of PBS containing various concentrations of tested agonists (naphazoline, clonidine, and DPMF) were added to each cell and then the solution was incubated for another 20 min at 37 °C. Reactions were stopped by removing tested regents and immediate adding ice-cold cell lysis buffer. For antagonist assays, the assays were carried out as described above, except that the respective 10 μM antagonists (yohimbine, mianserin, chlorpromazine, and phentolamine) were mixed with 10 μM of TA. The results are analyzed from three repeated experiments with standard deviations. All the data were processed using one site competitive binding, non-linear regression model in the GraphPad PRISM software version 6.0 (GraphPad). The 50% inhibition concentration (IC_50_) was obtained using the equation [16]: Y = (Top − Bottom)/(1 + 10^(X − LogIC_50_)) + Bottom, X: logmolar concentration of tested chemicals, Y: forskolin-stimulated cAMP levels, Top and Bottom: the maximum and minimum of cAMP level.

### 4.7. Data Analysis

All studies were replicated three to six times and plotted as mean ± standard errors using GraphPad Prism Software version 6.0 (GraphPad). Two-tailed, unpaired Student’s *t*-tests were used to test differences between two distributed groups of data. One-way analysis of variance (ANOVA) analyses paired with Tukey–Kramer post-hoc tests were used to test differences among multiple groups of data.

## 5. Conclusions

We cloned a gene for the tyramine receptor gene of *P. xylostella* (*Pxtar1*) and found that *tar1* mRNA levels in male adults were much higher than females (~30 fold higher), suggesting that *Pxtar1* may be involved in modulating the sexual behaviors of male organisms in this target pest species. Furthermore, we stably expressed *Pxtar1* in the HEK-293 cell line, and found PxTAR1 receptor was both highly specific to TA activation and was linked to the both endogenous calcium and cAMP pathways. Therefore, this receptor may be able to help us develop or screen insecticides used in the management of *P. xylostella*. Finally, we found that DPMF and L(-)-carvone can act on PxTAR1 as agonists, and yohimbine and mianserin can act as antagonists. Thus, these chemicals may represent useful tools for the investigation of the physiological and behavioral regulation of TAR1 in *P. xylostella*.

## Figures and Tables

**Table 1:** Primers used for *Pxtar1* gene clone and qPCR analysis.

Primer Name	Primer Sequences ^1^
TAR1_HindIII_F	TTTaagcttCTGCCACCATGGGGCAGGCCAACCT
TAR1_PstI_R	ATActgcagAGGCTTCATACAGAGCAGCTTC
TAR1_XbaI_F	GATtctagaGCCACCATGGGGCAGGCCAACCTC
TAR1_SalI_R	AGTgtcgacTCAAGGCTTCATACAGAGCAG
TAR1_qF	CTCGGTTGGAACGACTGGC
TAR1_qR	CGGTTCTGTCCGCTCGATAT
Rpl32_qF	TGCCCAACATTGGTTACGG
Rpl32_qR	ACGATGGCCTTGCGCTTC

^1^ Restriction enzyme recognition sites are printed in lower case.

## Supplementary Materials

Supplementary materials can be found at https://www.mdpi.com/1422-0067/20/12/2953/s1.

## Abbreviations

5-HT	Serotonin
AAs	Amino acids
(Ca^2+^)i	Intracellular Ca^2+^ concentration
(cAMP)i	Intracellular cyclic adenosine monophosphate concentration
CH	Chlorpromazine
DA	Dopamine
DPMF	N2-(2,4-Dimethylphenyl)-N1- methyformamidine
FROs	Female reproductive organs
GFP	Green fluorescent protein
MROs	Male reproductive organs
MS	Mianserin
OA	Octopamine
OARs	Octopamine receptors
ORF	Open reading frame
PA	Phentolamine
TA	Tyramine
TARs	Tyramine receptors
YHB	Yohimbine
