# Supplementary material for: Pharmacological Properties of the Type 1 Tyramine Receptor in the Diamondback Moth, Plutella xylostella
Source: Int J Mol Sci. 2019 Jun 17;20(12):2953. doi: 10.3390/ijms20122953 (PMC6627746; doi:10.3390/ijms20122953)
Supplement: Supplementary file 1 [file ijms-20-02953-s001.pdf]

1 **ATG**GGCAGGCCAACCTCACGATGGGCAACTCCACCTCACTGGACGCCGCGGCGGGCCCCGAGGAGTACTGCGCGGCGGCGGACGAGCCC

1 M G Q A N L T M G N S T S L D A A A G P E E Y C A A A D E P

91 CAGTACCCGAGCAGCCTCGGCCTGGCGCTGGCCGTGCCGCAGTGGGAGGCCGTCGCCACCGCCGTGGTCCTCACGCTCATCATCCTCTCG

31 Q Y P S S L G L A L A V P Q W E A V A T A V V L T L I I L S

181 ACCATCGTCGGGAACATCCTCGTCATCCTCAGCGTCTTCACCTACAAACCCCTGCGCATCGTCCAGAATTTCTTCATAGTTTCCCTCGCG

61 T I V G N I L V I L S V F T Y K P L R I V Q N F F I V S L A

271 GTCGCCGACCTCACCGTCGCCATACTGGTGCTGCCCTTCAACGTAGCCTACTCCATCCTCGGGCAGTGGGTGTTCGGCATCTACGTGTGC

91 V A D L T V A I L V L P F N V A Y S I L G Q W V F G I Y V C

361 AAGATGTGGCTCACGTGCGACATCATGTGCTGCACCTCGTCGATACTGAACCTGTGCGCCATCGCCCTCGACCGGTACTGGGCCATCACT

121 K M W L T C D I M C C T S S I L N L C A I A L D R Y W A I T

451 GACCCCATCAACTACGCGCAGAAGCGGACGCTCGAGCGAGTTCTGCTGATGATCGGCATCGTGTGGGCGCTGTCGCTCATCATCAGCTCG

151 D P I N Y A Q K R T L E R V L L M I G I V W A L S L I I S S

541 CCGCCGCTGCTCGGTTGGAACGACTGGCCCGAGGTCTTCGAGACCGACACGCCGTGCCGCCTCACCTCGCAGCCCGGCTTCGTCATCTTC

181 P P L L G W N D W P E V F E T D T P C R L T S Q P G F V I F

631 TCCTCCTCGGGATCCTTTTACATACCACTAGTTATTATGACCGTAGTTTATTTCGAAATTTACTTGGCCACCAAAAAGAGACTGAGGGAC

211 S S S G S F Y I P L V I M T V V Y F E I Y L A T K K R L R D

721 CGCGCCAAGGCAACTAAAATCAGCACCATATCGAGCGGACAGAACCGTCCGGCCAAAGACAACGACCACCACGACCAAGACTCGGTGAGT

241 R A K A T K I S T I S S G Q N R P A K D N D H H D Q D S V S

811 TCGGAGGCGAACCACAACGAGCACCCGGCGGCCGCGCGCCTTGTGGCCGACCCGCCCAAGAAACCCAGAAAGGCGACGCCAAAGAAGAGA

271 S E A N H N E H P A A A R L V A D P P K K P R K A T P K K R

901 CCGAAGAAGCGGTACTGGAGCAAGGACGACAAGTGCCACAACAAGTTGATCATTCCGATCCTGTCGAACGACAACTCGGTGACGGACGCG

301 P K K R Y W S K D D K C H N K L I I P I L S N D N S V T D A

991 GGCGAGGGGGAGGCGAGGCAGACGTCGTCGGAGAGCAACTCGAAGGAGGCGCATGAAGATGAGGTGATCCTGCGCGAGGCGAAGCCGCCG

331 G E G E A R Q T S S E S N S K E A H E D E V I L R E A K P P

1081 CCGAAGCCGAAGAGACCAAAGCCGCCGCAGCAGAACAGCGTGTACCAGTTCATCGAGGAGAAGCAGCGCATCTCGCTGACCCGCGAGCGG

361 P K P K R P K P P Q Q N S V Y Q F I E E K Q R I S L T R E R

1171 CGCGCGGCGCGGACGCTCGGCATCATCATGGGCGTGTTCGTGGTCTGCTGGCTGCCCTTCTTCGTCATCTACCTCGTCATCCCGTTCTGC

391 R A A R T L G I I M G V F V V C W L P F F V I Y L V I P F C

1261 GCCAGCTGCTGTCTCTCCAACAAGTTCATCAACTTCATCACGTGGCTCGGCTACGTCAACTCGGCACTCAACCCACTCATTTACACCATC

421 A S C C L S N K F I N F I T W L G Y V N S A L N P L I Y T I

1351 TTTAATATGGACTTCCGCAGAGCTTTCAAGAAGCTGCTCTGTATGAAGCCT**TAA**

451 F N M D F R R A F K K L L C M K P *

Figure S1. Nucleotide and deduced amino acid sequences of *P.xylostella* TAR1. Nucleotide (upper line) and amino acid (lower line) numbers are given on the left. Initial and termination codons are shown in bold.

Table S1. Similarity of predicted TAR1 protein from *P.xylostella* with orthologous proteins by BLAST analysis.

insects	Access ID	Protein name	Similarity
Bombyx mori	BAD11157	tyramine receptor	82%
Agrotis ipsilon	ACN12797	tyramine/octopamine receptor	83%
Spodoptera littoralis	ACJ06651	Oct/Tyr receptor	82%
Pieris rapae	AFX62896	tyramine receptor type 1	80%
Chilo suppressalis	AFG26689	TyR1	81%
Papilio xuthus	BAD72869	tyramine receptor	82%
Mythimna unipuncta	AFA55168	octopamine/tyramine receptor	87%
Trichoplusia ni	AFA55167	octopamine/tyramine receptor	85%
Danaus plexippus	EHJ68870	tyramine receptor	81%

Amino acid sequence alignment of PxTAR1 and orthologous receptors from GenBank

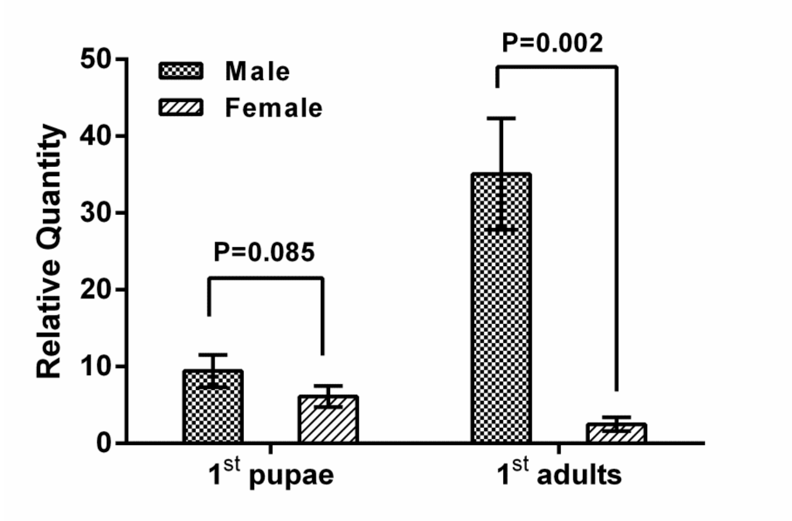

Figure S2. Expression of *Pxtar1* genes in different development stages (first day of pupae and first day of adults) of male and female in *P. xylostella*. Data represent means ±SE (n=3 repetitions) and were normalized to endogenous Rpl32, the internal control.

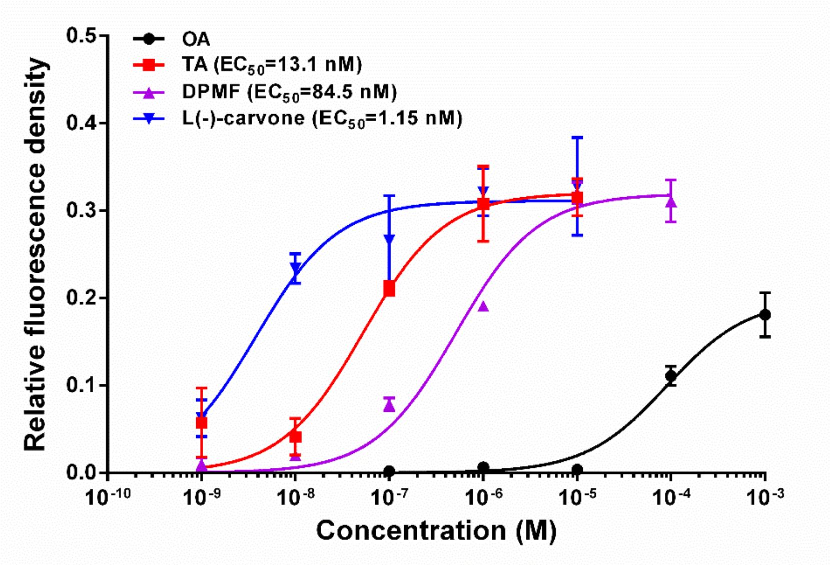

Figure S3. Effects of different agonists on $[Ca^{2+}]i$ in TAR1/293T cells. BioTek Synergy H1 Hybrid Multi-Mode Microplate Reader (excitation and detection wavelengths are 530 and 590 nm) were used to monitor the increase of intracellular calcium, using a calcium-sensitive fluorescent dye Cal-590™ AM, and maximum peak data in each assay were used for plotting. Data analyzed using GraphPad Prism EC anything (EC_{50}) [1]. Data is presented as the mean of three independent experiments. Relative fluorescence density was defined as: Real time fluorescence density / background fluorescence density.

[1] Gross, A.D.; Temeyer, K.B.; Day, T.A.; Perez de Leon, A.A.; Kimber, M.J.; Coats, J.R. Pharmacological characterization of a tyramine receptor from the southern cattle tick, *Rhipicephalus (Boophilus) microplus*. *Insect Biochem. Mol. Biol.* **2015**, *63*, 47–53, doi:10.1016/j.ibmb.2015.04.008.
